# Angiopoietin-1 and angiopoietin-2 are altered in polycystic ovarian syndrome (PCOS) during controlled ovarian stimulation

**DOI:** 10.1186/2045-824X-5-18

**Published:** 2013-10-24

**Authors:** Reshef Tal, David B Seifer, Richard V Grazi, Henry E Malter

**Affiliations:** 1 Genesis Fertility & Reproductive Medicine, Division of Reproductive Endocrinology and Infertility, Maimonides Medical Center, 1355 84th Street, Brooklyn, NY, USA

**Keywords:** Angiopoietin-1, Angiopoietin-2, Angiogenesis, Polycystic ovarian syndrome (PCOS), Controlled ovarian stimulation (COS)

## Abstract

Polycystic ovarian syndrome (PCOS) ovaries are characterized by increased angiogenesis and hypervascularity. While angiopoietin-1 (Ang-1) and its antagonist, angiopoietin-2 (Ang-2), are essential for ovarian function and angiogenesis, the levels of Ang-1 and Ang-2 in PCOS are unknown. This was a prospective cohort study of 14 PCOS women and 14 matched controls undergoing controlled ovarian stimulation (COS). Serum was collected on day 3, hCG and retrieval days. Follicular fluid (FF) was collected on retrieval day. Serum Ang-1 and Ang-2 levels were constant throughout COS, but serum Ang-1 levels were increased at all time points in PCOS women compared with controls (p < 0.05). No differences between groups were found in serum Ang-2 levels or FF Ang-1 levels. However, FF Ang-2 levels were increased almost 2-fold in PCOS women compared with controls (p < 0.01), and correlated positively with number of oocytes retrieved (r = 0.65, p < 0.0001). This study is the first to provide evidence of an alteration in the Ang-1/Ang-2 system in PCOS women. The biological role of Ang-2 in promoting capillary leakage, the increased Ang-2 FF level in PCOS, and its correlation with number of oocytes suggest that Ang-2 may play an important role in the increased risk of ovarian hyperstimulation in PCOS.

## Introduction

Polycystic ovarian syndrome (PCOS) affects between 5-7% of reproductive age women in Western societies. It is characterized by anovulatory infertility, hyperandrogenism, and polycystic ovaries [1,2]. In addition, women with PCOS are also at an increased risk of pregnancy complications, obesity, hyperlipidemia, insulin resistance, type II diabetes, and possibly cardiovascular disease [1,2].

Angiogenesis, blood vessel stabilization and regression within the ovary are critical components of the menstrual cycle [3]. Two of the most important classes of angiogenic factors, vascular endothelial growth factors (VEGFs) and the angiopoietins, are expressed in the ovary and regulate angiogenesis in regular follicular growth, ovulation, and the subsequent development and regression of the corpus luteum [4-7]. Ovaries of PCOS women are characterized by increased ovarian mass, supported by new blood vessel proliferation in the stroma and theca [8,9]. Consistent with increased angiogenesis, several studies have demonstrated increased expression of angiogenic growth factors in PCOS. VEGF has been shown to be upregulated in ovarian tissue of PCOS women [8] while both VEGF and basic fibroblast growth factor (bFGF) have been reported to be increased in serum and follicular fluid of PCOS women [9-11]. Moreover, soluble VEGF receptor-1 (sVEGFR-1), the circulating receptor for VEGF, has been shown to be decreased in serum of PCOS women undergoing controlled ovarian stimulation, contributing to increased VEGF bioavailability [12]. Taken together, these data suggest that PCOS patients have an angiogenic imbalance leading to a pro-angiogenic profile.

VEGFs and angiopoietins work in concert to control blood vessel formation, stabilization and regression [13]. Angiopoietin-1 (Ang-1) and angiopoietin-2 (Ang-2) act on vascular endothelial cells via the same tyrosine kinase receptor, Tie-2. However, they exert opposing effects following receptor binding. Ang-1 recruits and interacts with perivascular smooth muscle cells (pericytes) stabilizing the neovessels formed by the action of VEGF [14]. In contrast, Ang-2 is a natural antagonist for Ang-1 and plays a role in loosening the support of extracellular cell matrix and destabilization of existing blood vessels by antagonizing the action of Ang-1 [15]. It is thought that in the presence of VEGF, the action of Ang-2 promotes angiogenesis by allowing endothelial cell proliferation and migration, whereas in the absence of VEGF, Ang-2 signal blocks the recruitment of pericytes, leading to vessel destabilization and regression [13,16]. Ang-1, Ang-2 and their Tie-2 receptor are expressed in the ovarian follicle and corpus luteum and have been shown to play a critical role in follicular growth, ovulation and corpus luteum development in both primates and humans [4-6]. In fact, disruption of the Ang-1/Ang-2 balance by intrafollicular Ang-2 injection prevents follicular angiogenesis, ovulation and subsequent corpus luteum formation in primates [17].

Recently, Ang-1 and Ang-2 serum and follicular fluid levels have been reported in humans during controlled ovarian stimulation [18]. In addition, serum Ang-2 levels in non-stimulated women were not found to be different between PCOS and matched controls [19]. However, despite evidence suggesting an important role for angiopoietins in ovarian angiogenesis, their levels and dynamics have not been previously investigated in PCOS during controlled ovarian stimulation. Previously, we reported that the proangiogenic transforming growth factor-β (TGF-β) is increased while its soluble endoglin receptor is decreased in serum of PCOS women, contributing to greater TGF-β bioavailability and proangiogenic state [20]. We hypothesized that Ang-1 and Ang-2 levels may be increased in PCOS, consistent with increased angiogenesis and hypervascularity observed in PCOS ovaries. Accordingly, we wished to investigate Ang-1 and Ang-2 levels and temporal expression patterns in serum and follicular fluid of PCOS women as compared to non-PCOS control women during controlled ovarian stimulation in our previously described study population [20].

## Methods

### Study subjects

We prospectively enrolled 14 PCOS women from women undergoing controlled ovarian stimulation (COS) in preparation for IVF or ICSI as previously described [20]. Diagnosis of PCOS was made according to the Rotterdam consensus [21]. All 14 PCOS women had oligo- or amenorrhea and at least 12 follicles 2-9 mm in diameter per ovary. Ten of the women had hyperandrogenemia and/or hyperandrogenism, and four had normal serum androgen levels and no clinical hyperandrogenism. Secondary causes of androgen excess and anovulation were excluded. The indications for COS in the PCOS group were male factor (7 women), ovulatory dysfunction after failed superovulation induction and intrauterine inseminations (4 women) and tubal factor (3 women). Fourteen non-PCOS control women undergoing COS in preparation for IVF or ICSI were matched to PCOS women by age, BMI and use of GnRH agonist/antagonist prior to enrollment. Inclusion criteria for control non-PCOS women were normal ultrasonic ovarian morphology, normal ovulatory cycles and no endocrine abnormalities. Inclusion criteria for all study women were ages between 20–38, adequate visualization of ovaries on transvaginal ultrasound and no hormonal treatment i.e. OCPs. Exclusion criteria were diminished ovarian reserve or endometriosis. The indications for COS in the control group were male factor (9 women) or tubal factor infertility (5 women). Patient clinical data is given in Table 1. This study was approved by the Maimonides Medical Center institutional review board. Informed consent was obtained from all participating women.

**Table 1:** Patient clinical data

	**PCOS**	**Non-PCOS**	**P value**
Age (years)	30.1 ± 4.4	30.8 ± 3.7	NS
Body mass index (Kg/m^2^)	25.5 ± 5.6	24.9 ± 3.9	NS
Serum AMH (ng/ml)	8.0 ± 6.5	3.1 ± 1.5	0.003
Day 3 FSH (mIU/ml)	5.2 ± 1.9	6.0 ± 2.7	NS
Total gonadotropin dose (IU)	2057 ± 868	2767 ± 1786	NS
Estrogen on day of hCG (pg/ml)	2819 ± 1158	2898 ± 860	NS
No. of aspirated oocytes	17.4 ± 10.5	12.6 ± 7.2	NS
OHSS (%)	3/14 (21.4)	0/14 (0)	NS

Note: PCOS, polycystic ovarian syndrome; OHSS, ovarian hyperstimulation syndrome; NS, not significant; values are mean ± standard deviation. P-value < 0.05 was considered statistically significant.

Women were treated using a stimulation protocol which included either down regulation using a GnRH agonist in a long protocol (n=8 for each group) or a GnRH antagonist to prevent premature ovulation (n=6 for each group). Ovarian stimulation was performed using a combination of recombinant FSH and HMG. The standard stimulation protocol was modified when there was risk of ovarian hyperstimulation or previous history of poor response. Follicular monitoring by ultrasound and blood sampling for estradiol levels were performed every 1–3 days. After the first 3–5 treatment days, the daily dose could be adjusted based on the follicular development and estradiol levels. When at least six follicles with a diameter of 16 mm were detected, either 5,000 or 10,000 IU hCG was administered, depending on the estimated risk for hyperstimulation. Oocytes were retrieved under transvaginal sonographic guided needle puncture 35 hours following hCG administration.

### Collection of blood and follicular fluid

Blood samples were obtained by venipuncture on cycle day 3, day of hCG administration and day of oocyte retrieval. After collection, the blood samples were allowed to clot at room temperature for 30 min, followed by centrifugation at 1200 rpm for 10 min. Serum was stored in aliquots at -80°C until assayed. For follicular fluid collection, follicles with a diameter of >16 mm were aspirated. Only the first clear follicular fluid aspirate associated with the presence of an oocyte, without blood or flushing solution, was used for analysis. After removal of the oocyte, the fluid was centrifuged at 1200 rpm for 10 min to remove granulosa cells and debris. The supernatant was divided into aliquots and stored at -80°C until assayed.

### Ang-1, Ang-2 and AMH ELISA assays

Ang-1 and Ang-2 concentrations of serum and follicular fluid samples were determined by ELISA according to the manufacturer’s protocols (R&D, Minneapolis, MN), having a sensitivity of 62.5 pg/mL and 46.9 pg/mL, respectively. The intra- and inter-assay coefficients of variation for Ang-1 and Ang-2 ELISAs were 2.4% and 5.5%, and 6.5% and 9.1%, respectively. AMH concentration of serum and follicular fluid samples was determined according to the manufacturer’s protocols (Beckman and Coulter, Brea, CA). The assay sensitivity was 0.16 ng/ml. The intra- and inter-assay coefficients of variation were 4.3% and 7.4%, respectively. All assays were performed in duplicate.

### Statistics

A power analysis powered for a two-tailed t-test was carried out prior to subject enrollment to determine the minimum required sample sizes needed to detect a 1.5-fold (assumed) statistically significant difference in growth factor between cases (PCOS) and controls. The required level of significance was at least 95% (α=0.05), and the required power of the test was at least 90% (β=0.10). Based on this calculation the required sample sizes were n = 14 for each group. Data were analyzed by Student’s t-test or the Mann–Whitney test, as appropriate. Results are expressed as mean ± standard error of the mean (SEM). Correlations between angiopoietins and number of oocytes retrieved were performed using Pearson correlation tests. SigmaStat (SPSS Science, Chicago, IL) was used for statistical analysis. All significance tests were two-tailed and P-value < 0.05 was considered to be statistically significant.

## Results

There were no differences between PCOS and non-PCOS women in terms of age, body mass index, day 3 FSH, total amount of gonadotropins administered, estrogen levels on day of hCG administration and number of aspirated oocytes (Table 1). In contrast, as expected, PCOS women had a significantly higher serum AMH level than the non-PCOS control group (8.0 ± 6.5 ng/ml vs. 3.1 ± 1.5 ng/ml, p = 0.003) (Table 1). In addition, 3 of 14 PCOS women developed mild OHSS whereas no OHSS was noted in the non-PCOS group.

**Figure 1:**
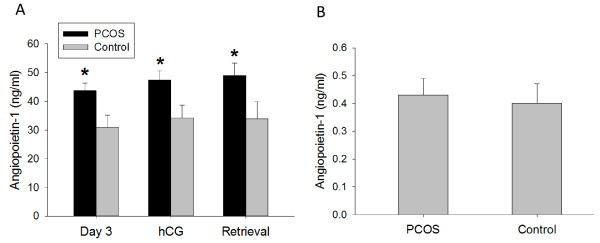
**Ang-1 concentration in (A) serum (ng/ml) and (B) follicular fluid (ng/ml) of PCOS (polycystic ovarian syndrome) and non-PCOS women undergoing controlled ovarian stimulation.** Serum Ang-1 levels were consistently increased in PCOS compared with non-PCOS women throughout controlled ovarian stimulation, while no differences between groups were observed in follicular fluid. Data are presented as mean ± SEM. *p < 0.05 for PCOS vs. non-PCOS women.

Ang-1 serum levels did not change throughout controlled ovarian stimulation in either PCOS or control women (Figure 1A). However, Ang-1 serum levels were significantly greater in PCOS women compared with non-PCOS controls at all three time points, on day 3 (43.7 ± 2.6 ng/ml vs. 30.9 ± 4.3 ng/ml, p = 0.02), day of hCG administration (47.4 ± 3.1 ng/ml vs. 34.2 ± 4.5 ng/ml, p = 0.02), and day of oocyte retrieval (49.0 ± 4.3 ng/ml vs. 33.9 ± 6.0 ng/ml, p = 0.04) (Figure 1A). No differences between groups were observed in follicular fluid Ang-1 levels (Figure 1B). In addition, no correlation was found between serum or follicular fluid Ang-1 levels and number of oocytes retrieved (data not shown).

**Figure 2:**
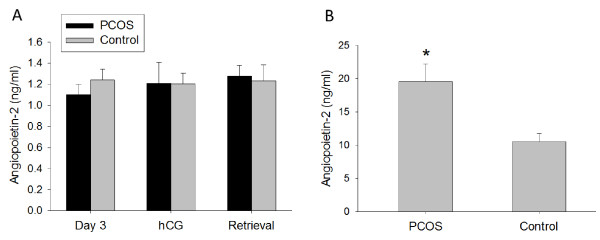
**Ang-2 concentration in (A) serum (ng/ml) and (B) follicular fluid (ng/ml) of PCOS (polycystic ovarian syndrome) and non-PCOS women undergoing controlled ovarian stimulation.** Follicular fluid Ang-2 levels were increased in PCOS compared with non-PCOS women. No differences between groups were noted in serum Ang-2 levels. Data are presented as mean ± SEM. *p < 0.01 for PCOS vs. non-PCOS women.

**Figure 3:**
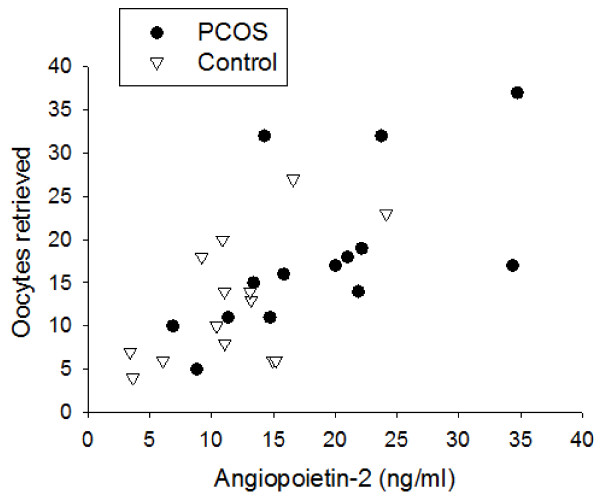
Correlation between follicular fluid Ang-2 levels and total number of oocytes retrieved in women with PCOS and non-PCOS controls, demonstrating a significant positive correlation in both groups (r = 0.65, p < 0.0001).

Similarly to Ang-1, Ang-2 serum levels were constant throughout controlled ovarian stimulation in either PCOS or non-PCOS women (Figure 2A). In contrast to Ang-1 serum levels, Ang-2 serum levels were not different between the two groups on day 3, day of hCG or day of oocyte retrieval (Figure 2A). Furthermore, follicular fluid Ang-2 levels were 1.9-fold greater in PCOS women compared with non-PCOS controls (19.6 ± 2.6 ng/ml vs. 10.5 ± 1.2 ng/ml, p < 0.01) (Figure 2B). Moreover, Ang-2 levels in follicular fluid were found to positively correlate with the number of oocytes retrieved in both PCOS and non-PCOS groups (r = 0.65, p < 0.0001) (Figure 3). No correlation was noted between serum Ang-2 levels and number of oocytes retrieved (data not shown).

Ang-1 concentration was markedly higher in serum compared with follicular fluid, with serum/follicular fluid ratios ranging from 102–114 and 77–85 in PCOS and non-PCOS groups, respectively (data not shown). Conversely, Ang-2 levels were greater in follicular fluid than serum, with a reversal in serum/follicular fluid ratio, ranging from 0.06-0.07 and 0.11-0.12 in PCOS and non-PCOS groups, respectively (data not shown). Since Ang-1 and Ang-2 exert opposing actions, their ratio may provide insight into the angiogenic status, and particularly the propensity for vessel stabilization versus regression. The Ang-1/Ang-2 ratio in serum of PCOS women was significantly increased compared with non-PCOS women at all three measured time points, on day 3 (39.7 ± 2.3 vs. 24.9 ± 2.6, p = 0.02), day of hCG administration (39.2 ± 2.9 vs. 28.5 ± 1.7, p = 0.04), and day of oocyte retrieval (38.2 ± 3.1 vs. 27.3 ± 1.9, p = 0.01) (Table 2). Remarkably, the Ang-1/Ang-2 ratio was reversed in follicular fluid compared with serum, due to lower Ang-1 and higher Ang-2 levels in follicular fluid compared with serum. In addition, the Ang-1/Ang-2 ratio was significantly decreased in follicular fluid of PCOS compared with non-PCOS women (0.02 ± 0.004 vs. 0.04 ± 0.006, p = 0.002), consistent with increased follicular fluid Ang-2 levels in the PCOS group (Table 2).

**Table 2:** Ang-1/Ang-2 ratio in serum and follicular fluid of PCOS and non-PCOS women

**Sample**	**PCOS**	**Non-PCOS**	**P value**
Serum day 3	39.7 ± 2.3	24.9 ± 2.6	0.02
Serum day of hCG	39.2 ± 2.9	28.5 ± 1.7	0.04
Serum day of retrieval	38.2 ± 3.1	27.3 ± 1.9	0.01
Follicular fluid	0.02 ± 0.004	0.04 ± 0.006	0.002

Note: PCOS, polycystic ovarian syndrome; Data are presented as mean ± S.E.M.

## Discussion

This is the first study to report levels and dynamics of Ang-1 and Ang-2 in PCOS women during the course of controlled ovarian stimulation. Our data demonstrate that serum Ang-1 and Ang-2 levels remained unchanged during controlled ovarian stimulation in both PCOS and non-PCOS women. However, Ang-1 levels were found to be increased in serum of PCOS compared with non-PCOS women throughout controlled ovarian stimulation, while Ang-2 was increased in follicular fluid of PCOS women compared with controls. Consequently, Ang-1/Ang-2 ratio was increased in serum but decreased in follicular fluid of PCOS relative to non-PCOS women. Moreover, this study is the first to report a correlation between follicular fluid Ang-2 levels and number of oocytes retrieved, a finding which may have important clinical implications for IVF and ovarian hyperstimulation syndrome (OHSS).

In the present study, serum Ang-1 levels were unchanged during controlled ovarian stimulation but were consistently increased in PCOS women compared with non-PCOS controls. Serum Ang-2 levels were also unchanged throughout controlled ovarian stimulation. However, in contrast to Ang-1, no differences were noted in serum Ang-2 levels between PCOS and non-PCOS women. Our data are consistent with a previous report that did not find a difference in serum Ang-2 levels between unstimulated PCOS and matched non-PCOS women [19]. Elevated serum Ang-1 in PCOS in our study is also consistent with several previous reports indicating increased angiogenesis in PCOS women. VEGF has been shown to be increased in serum [9,11] and ovarian tissue [8] of PCOS women compared with non-PCOS controls. Moreover, VEGF and basic fibroblast growth factor (bFGF) levels have been shown to be greater in serum and follicular fluid of PCOS women compared to controls [10]. These data suggest that PCOS patients have an angiogenic dysregulation promoting angiogenesis. Ang-1 is widely expressed mainly by smooth muscle cells, adventitial cells and endothelial cells of the vasculature [22]. Since the concentration of Ang-1 is much greater in serum compared to follicular fluid, the systemic vascular bed is the likely source of increased circulating Ang-1 levels in PCOS and not the ovary.

In the current study, Ang-1 serum levels during controlled ovarian stimulation were 77 to 114-fold greater than follicular fluid levels. In contrast, Ang-2 serum levels during controlled ovarian stimulation were 8 to 16-fold lower than follicular fluid levels. Accordingly, the Ang-1/Ang-2 ratio in serum ranged from 24.9 to 39.7, while Ang-1/Ang-2 ratio in follicular fluid was reversed, ranging from 0.02 to 0.04. Follicular fluid is the product of diffusion of blood constituents across the blood-follicle barrier and of molecules secreted by granulosa and theca cells. Our data suggest that the local ovarian angiopoietin milieu is not reflected in the bloodstream and that Ang-2 is produced in large amount locally within the ovary and is the predominant angiopoietin in this microenvironment. The great difference in Ang-1 concentration between serum and follicular fluid may be explained by the propensity of Ang-1 to incorporate into and be sequestered by extracellular matrix, making it unlikely to diffuse to distant organs, in contrast to Ang-2 [23]. In addition, Ang-1, a 70 kDa molecule, predominantly exists in high-order multimers [24], which may be speculated to limit its diffusion capacity across the blood-follicle barrier. Our data support earlier findings by Hurliman et al. who reported Ang-1/Ang-2 ratios in serum and follicular fluid during controlled ovarian stimulation in a population of oocyte donors [18]. They found similar Ang-1/Ang-2 ratios in serum and follicular fluid to the ones reported in our study. In addition, consistent with our results, Hurliman et al. did not find changes in serum Ang-1 or Ang-2 levels throughout controlled ovarian stimulation or menstrual cycle [18]. In contrast to the lack of variation in Ang-1 and Ang-2 levels in serum throughout follicular growth, selection, ovulation and luteinization, marked changes in Ang-1 and Ang-2 levels occur in the ovaries during the human menstrual cycle [5,6], further supporting the notion that local ovarian angiopoietin levels are not reflected in the bloodstream. Moreover, the lack of change in Ang-1 and Ang-2 serum levels following oophorectomy is consistent with this premise [18].

Our data and that of Hurliman et al. [18] showed that following controlled ovarian stimulation Ang-1/Ang-2 ratio in follicular fluid is very low, consistent with ovarian predominance of Ang-2 over Ang-1 in the periovulatory period. Evidence from several studies is consistent with this hypothesis. Wulff et al. have reported that ratio of Ang-2/Ang-1 ovarian mRNA expression ranges from 2 to 47-fold during the menstrual cycle, reaching the greatest ratio following hCG injection [5]. Similarly, Sugino et al. have shown that ovarian Ang-2 mRNA and protein levels are significantly greater than Ang-1 throughout the menstrual cycle [6]. Moreover, they showed that ovarian Ang-2 levels are greatest during the follicular and early luteal phase, while Ang-1 levels are lowest during the early luteal phase [6]. The proposed Ang-2 predominance in the ovary in the periovulatory period may have important implications for the pathogenesis of ovarian hyperstimulation (OHSS).

VEGF is a key mediator of OHSS and is found to be elevated in the circulation [25,26], follicular fluid [27] and ascites fluid [28] of patients developing this syndrome. Its dysregulation in PCOS has been suggested to contribute to the increased risk of OHSS seen in these patients [29]. VEGF is thought to be secreted by the ovarian endothelial and granulosa cells and promote vascular permeability leading to significant fluid extravasation in OHSS [29]. The agonist Ang-1 and its antagonist Ang-2 are also known to influence vascular permeability in the context of angiogenesis. Ang-1 stabilizes newly formed vessels and reduces capillary permeability and leakage [14,30]. In contrast, the action of Ang-2 has been shown to antagonize Ang-1 [15] and lead to vessel destabilization and increased permeability [31,32]. In the current study, PCOS women had significantly greater Ang-2 levels in follicular fluid than non-PCOS women following controlled ovarian stimulation. This resulted in lower Ang-1/Ang-2 follicular fluid ratio in PCOS compared with non-PCOS women, indicating that the follicular microvasculature in PCOS may be more prone to fluid leakage. These data suggest that the increased risk for OHSS development in PCOS women may be explained, at least in part, by Ang-2 overactivity. Our data demonstrating a strong correlation between follicular fluid Ang-2 levels and the number of oocytes retrieved i.e. ovarian stimulation provide further support for this notion. In our study, 3 of 14 PCOS women developed mild OHSS while no OHSS was noted in the control group. Ang-1 and Ang-2 serum and follicular fluid levels were not different between patients who developed OHSS compared to those who did not (data not shown). However, our study is limited by small number of patients and was not powered for such a comparison. Future studies are warranted to evaluate the potential role of Ang-2 in OHSS pathogenesis and examine its utility as a predictor for early and/or late OHSS. Such studies may pave the way for better monitoring and individualization of ovarian stimulation for those patients who are at highest risk of OHSS, in addition to potential development of new biological interventions targeting the angiopoietin system for prevention and/or treatment of OHSS.

In conclusion, this is the first study to characterize Ang-1 and Ang-2 levels in PCOS during controlled ovarian stimulation and to provide evidence of an alteration in the Ang-1/Ang-2 system in PCOS women compared with control women. The concentrations of serum Ang-1 and Ang-2 are relatively constant throughout controlled ovarian stimulation, but serum Ang-1 levels are consistently increased in PCOS women compared with controls. In addition, the predominance of Ang-2 over Ang-1 in the ovary, increased Ang-2 follicular fluid levels in PCOS, and the correlation of follicular fluid Ang-2 levels with number of oocytes retrieved suggest a potential role for Ang-2 in the angiogenic dysregulation seen in PCOS women, and their predisposition for OHSS development. Further studies are indicated to explore these possibilities.

## Competing interest

D.B.S. receives royalties from a licensing agreement between UMDNJ/MGH and Beckman Coulter for the use of MIS/AMH in determining ovarian reserve. R.T., R.V.G. and H.E.M have nothing to declare.

## Authors’ contribution

RT conceived and designed the study, performed the ELISA experiments and statistical analysis, and drafted the manuscript. DBS designed the study and edited the manuscript. RVG edited the manuscript. HEM helped with study design and performed patient sample collection. All authors read and approved the final manuscript.
